# Genetic Polymorphisms as Key Modulators of Cardiovascular Risk from Endocrine-Disrupting Chemicals

**DOI:** 10.3390/genes17060717

**Published:** 2026-06-21

**Authors:** Mariangela Palazzo, Francesca Gorini, Ludovica Simonini, Fabrizio Minichilli, Andrea Borghini

**Affiliations:** Institute of Clinical Physiology, National Research Council, 56124 Pisa, Italy; mariangelapalazzo@cnr.it (M.P.); ludovicasimonini@cnr.it (L.S.); fabrizio.minichilli@cnr.it (F.M.)

**Keywords:** endocrine-disrupting chemicals, PFAS, PCBs, PBDEs, PAHs, dioxins, phthalates, bisphenols, cardiovascular risk, genetic polymorphisms

## Abstract

Environmental exposure to persistent and non-persistent endocrine-disrupting chemicals (EDCs), including per- and polyfluoroalkyl substances (PFAS), polychlorinated biphenyls (PCBs), polybrominated diphenyl ethers (PBDEs), polycyclic aromatic hydrocarbons (PAHs), dioxins, phthalates, and bisphenols, has been increasingly associated with elevated cardiovascular disease (CVD) risk. Emerging evidence suggests the importance of gene–environment interactions in modulating individual susceptibility to EDC-related cardiovascular effects. This review summarizes current knowledge by synthesizing the main classes of EDCs, evaluating the evidence linking them to cardiovascular outcomes, and highlighting how genetic variability may modulate EDC-induced cardiovascular risk. Across the studies analyzed, the most extensively investigated genetic polymorphisms involve pathways related to oxidative stress regulation, xenobiotic metabolism and detoxification, hormone signaling, and lipid homeostasis. Variants in antioxidant defense genes, such as *CAT*, *eNOS*, and *PON1*, have been associated with increased hypertension risk and vascular dysfunction following exposure to bisphenols and PAHs. Polymorphisms in *GSTP1*, *CYP2C19*, CYP1A2, CYP2E1, *ABCB1*, and *MTHFR* may influence susceptibility to cardiometabolic alterations and congenital heart defects, whereas variants in *ESR2*, *FTO*, *LEPR*, and *INSIG2* have been linked to obesity, dyslipidemia, and hypertension associated with PFAS, PBDEs, and bisphenols. A deeper understanding of gene–environment interactions is essential to advance preventive cardiology and mitigate the cardiovascular impact of environmental pollutants.

## 1. Introduction

Endocrine-disrupting chemicals (EDCs), defined as naturally occurring substances or synthetically produced chemicals capable of altering the endocrine system and consequently causing adverse health effects in intact organisms-both wildlife and humans-have progressively become a global public health concern over recent years owing to the rapid expansion of modern industrial activities and the consequent release of these pollutants into the environment [1,2,3]. In addition to EDCs commonly encountered in everyday life through food contact materials, plastic bottles, cosmetics, detergents, and toys (bisphenols, phthalates), other EDCs belong to the class of persistent organic pollutants (POPs) [4]. Compounds such as polychlorobiphenyls (PCBs), polybrominated diphenyl ethers (PBDEs) and per- and polyfluoroalkyl substances (PFAS) are intentionally designed for industrial purposes to exhibit long environmental persistence and are characterized by high lipophilicity, resistance to chemical degradation, and a strong tendency to bioaccumulate in ecosystems and in the adipose tissue of organisms, with persistence extending up to several decades [5,6]. Other POPs including polycyclic aromatic hydrocarbons (PAHs) and dioxins, are not intentionally manufactured but are generated mainly as byproducts of incomplete combustion of organic materials, particularly during industrial processes [3,7]. The list of substances identified as EDCs is continuously evolving, and it has been estimated that close to one thousand compounds have been characterized as EDCs among the tens of thousands of synthetic chemicals currently detected in the environment [4,5].

Importantly, these substances may exert biological effects at low doses, referring to EDC concentrations that are relevant to human exposure levels, and, similar to endogenous hormones, can act through non-monotonic dose–response relationships [8]. Although human exposure to EDCs occurs predominantly via dietary intake and, to a lesser extent, through dermal contact and inhalation, certain classes of EDCs—such as plasticizers and flame retardants—are capable of transplacental transfer, accumulate in placental tissues, and persist in the fetal circulatory system and organs, thereby posing potential risks to fetal development [4,6].

EDCs can adversely affect cardiovascular health by altering hormonal regulation through multiple mechanisms, even as early as fetal life, when EDCs may interfere with developmental processes [9,10]. Within the developmental origins of health and disease (DOHaD) framework, early-life exposure to certain EDCs such as phthalates and bisphenols can “program” long-term cardiovascular risk, increasing susceptibility to perinatal and postnatal cardiometabolic outcomes [11]. This is particularly relevant given that CVD remains the leading cause of global mortality, with rising incidence partly driven by environmental exposures [12,13]. Epidemiological evidence links EDCs such as phthalates, bisphenols, PCBs, PFAS, and PAHs to adverse cardiovascular outcomes and risk markers, including elevated blood pressure, altered blood lipid profile, and atherosclerosis [14,15].

Individual vulnerability to these effects is further modulated by genetic variability, which influences both the magnitude and progression of CVD in response to EDC exposure. Genetic variants in genes involved in xenobiotic metabolism, oxidative stress, lipid and homocysteine metabolism, and hormone signaling-including GSTM1, GSTT1, CYP enzymes, MTHFR, PON1, PPARG, and estrogen receptor genes-may modify susceptibility to the cardiovascular and cardiometabolic effects of EDCs [16,17,18,19,20,21,22]. Although single nucleotide polymorphisms (SNPs) provide important insights into genetic susceptibility, further research is needed to clarify the mechanisms through which they influence responses to EDCs. Establishing SNPs as reliable predictors of disease risk is essential for improving personalized risk assessment and guiding preventive and therapeutic strategies. However, identifying the most relevant genetic determinants in EDC-related disease pathways remains challenging due to the complexity of gene–environment interactions.

This review aims to synthesize current evidence on the impact of SNPs on biological responses to EDC exposure, including PCBs, PBDEs, PFAS, PAHs, dioxins, phthalates, and bisphenols. By examining gene–environment interactions in CVD development, the review discusses key underlying biological mechanisms. It also explores how individual genetic profiles may modulate responses to EDC exposure, offering potential targets for personalized prevention and treatment strategies.

## 2. Human Biomonitoring and Cardiovascular Effects of Endocrine-Disrupting Chemicals

EDCs have been detected in all ecosystems tested, even those located in remote places, with surface waters as the most contaminated media [2]. These contaminants undergo bioaccumulation, bioconcentration, biomagnification, and long-range transport through food webs and water cycles, ultimately reaching humans as final consumers and affecting all life stages [23]. As EDCs are present in a wide range of everyday products, humans are often exposed to multiple chemicals simultaneously [24]. Human biological biomonitoring (HBM) is a key approach for assessing internal exposure, as it reflects the real-life chemical body burden of contaminants and/or their metabolites resulting from total exposure to chemical substances from multiple sources and through different routes [25]. Therefore, HBM can provide essential information not only on total exposure to contaminants, including EDCs, and on how exposure patterns change over time or differ across geographical regions and populations, but also on the effectiveness of policy actions [25].

**Figure 1:**
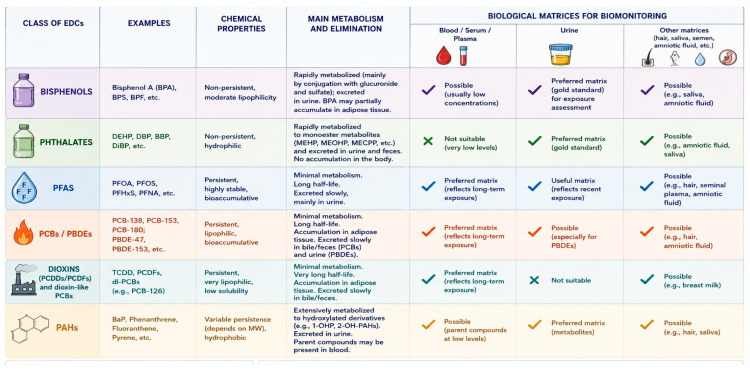
Human biomonitoring strategies for the assessment of major classes of endocrine-disrupting chemicals (EDCs) in biological matrices. The figure summarizes the main chemical classes of EDCs, including bisphenols, phthalates, per- and polyfluoroalkyl substances (PFAS), polychlorinated biphenyls (PCBs), polybrominated diphenyl ethers (PBDEs), dioxins, and polycyclic aromatic hydrocarbons (PAHs), together with their physicochemical properties, metabolic fate, and preferred biological matrices for exposure assessment. Persistent and lipophilic compounds, such as PFAS, PCBs/PBDEs, and dioxins, are primarily measured in blood, serum, or plasma because they bioaccumulate and reflect long-term exposure. In contrast, non-persistent and rapidly metabolized compounds, including bisphenols, phthalates, and PAH metabolites, are preferentially quantified in urine. Alternative matrices such as hair, saliva, semen, breast milk, and amniotic fluid may provide complementary exposure information in selected contexts. The figure also highlights the importance of repeated sampling or pooled specimens for accurately assessing exposure to non-persistent EDCs characterized by high intra-individual variability. Image generated with ChatGPT-v5.5.

HBM typically assesses the internal concentrations of EDCs and/or their metabolites in blood or urine specimens, although other biological matrices such as amniotic fluid, hair, saliva, and semen can also be used [26]. In general, persistent chemicals (e.g., PCBs, PBDEs, PFAS) are commonly measured in blood or blood derivatives, whereas non-persistent EDCs (e.g., phthalates, bisphenols), due to their hydrophilic nature, undergo extensive metabolism and are conjugated mainly with glucuronide and sulfate groups through human detoxification pathways [24,27]. These reactions generate stable metabolites in varying proportions, which are ultimately excreted via urine [24]. Importantly, while a single concentration of persistent EDCs is a reliable biomarker of exposure over time-regardless of the duration and intensity of exposure-for non-persistent EDCs a single-sample measurement cannot adequately reflect true exposure [27]. Their concentration fluctuates markedly depending on the timing, duration, and intensity of exposure; therefore, multiple measurements or the use of pooled specimens is required to provide a more accurate estimate [27] (Figure 1).

Over the past decade, human biomonitoring of EDCs has gained increasing relevance, also in relation to their potential impact on cardiovascular health. However, despite the growing body of research, the association between EDC exposure and cardiovascular risk remains somewhat inconsistent [28]. Among non-persistent EDCs, bisphenol A (BPA) is a high–production-volume chemical, with approximately ten million tons produced annually worldwide [29]. It is widely used as a monomer in the manufacture of polycarbonate plastics and epoxy resins, which are employed in metal can linings, food containers, baby bottles, dental sealants, medical equipment, thermal paper, toys, and numerous other consumer products [26,30]. Notably, BPA may partially accumulate in adipose tissue, which could account for the relatively slow decline of BPA levels in urine [31]. While one meta-analysis including data from six National Health and Nutrition Examination Survey (NHANES) examination cycles found no significant associations between urinary BPA and five lipid variables related to cardiovascular risk [30], the meta-analysis by Fu et al., which included a total of ten studies, reported a pooled odds ratio (OR) of 1.19 (95% Confidence Interval–95%CI: 1.03–1.37) for the association between BPA exposure and CVD risk factors [28]. Furthermore, a cross-sectional study based on data from NHANES 2003–2012, demonstrated a significantly positive association between BPA and the risk of CVD (defined as a composite of five self-reported outcomes), with an OR = 1.09 (95%CI: 1.01–1.18, *p* < 0.05) per one-unit increment in log-transformed urinary BPA and after adjusting for all covariates [32] These findings support previous results by Yin et al., who reported significant positive associations between urinary BPA levels and both total and individual CVD outcomes, including angina, congestive heart failure, and stroke [33].

Phthalates are the most widely used plasticizers, with a broad range of applications in both industrial and household products [26]. Current global production is estimated at approximately 11 million tons, with continued growth driven by low production costs and the lack of affordable alternatives [34]. In contrast to BPA, phthalates are rapidly metabolized to monoester metabolites and excreted in urine and feces, without accumulating in the human body [26]. Nevertheless, phthalates appear to affect cardiovascular health. In a meta-analysis including five studies that evaluated the association between urinary levels of eight phthalate metabolites and CVD risk, no significant associations were observed for individual metabolites; however, the pooled OR for total phthalate exposure was 1.11 (95%CI: 1.06–1.17) [28]. A recent epidemiological analysis using data from NHANES 2005–2018 reported a significant positive association between phthalate mixtures and CVD prevalence (OR = 1.21, 95%CI: 1.07–1.37, *p* = 0.002) [35]. The study identified several metabolites—mono-(2-ethyl-5-oxohexyl) phthalate (MEOHP), mono-2-ethyl-5-carboxypentyl phthalate (MECPP), monobenzyl phthalate (MBzP), and mono-*n*-butyl phthalate (MnBP)—as independent and key predictors of CVD (*p* < 0.05) [35].

PFAS are a class of fluorinated synthetic persistent organic chemicals known for their water-resistant, stain-resistant, fire-resistant, and non-stick properties [36,37]. They are produced in high volumes and widely used in numerous industrial and consumer applications, including food-contact materials, personal care products, firefighting foams, and textile protective coatings, resulting in widespread contamination of all environmental matrices [36,38]. Despite the well-established association between PFAS exposure and elevated cholesterol levels in both mechanistic and epidemiological studies, evidence supporting a link with CVD remains limited [39,40]. Two recent Swedish longitudinal studies found no significant association between plasma PFAS levels and incident CVD; one assessed perfluorooctanoic acid (PFOA), perfluorooctane sulfonic acid (PFOS,) and perfluorohexanesulfonic acid (PFHxS) in middle-aged individuals, while the other evaluated six PFAS in elderly participants, using a composite endpoint of myocardial infarction, ischemic stroke, and heart failure [36]. Furthermore, a meta-analysis of five independent studies reported a significant inverse association between PFOA levels and incident CVD risk for highest vs. lowest exposure (Relative Risk, RR = 0.80; 95% CI: 0.66–0.94) [36].

PCBs and PBDEs are two families of POPs in which variation in the number and position of chlorine or bromine atoms gives rise to 209 distinct congeners, resulting in differences in chemical properties and potential toxicity [41,42,43]. Among PCBs, non-coplanar congeners exhibit high reactivity and toxicity, with PCB-77 being one of the most toxic, whereas among PBDEs, PBDE-47 is considered one of the most biologically potent congeners [41]. PCBs have been used as flame retardants and plasticizers [41]; however, their production has been banned in most countries worldwide following ratification of the Stockholm Convention [44]. PBDEs, used since the 1970s in paints, plastics, textiles, building materials, aircraft, and automobiles, were banned in most countries in the early 2000s (penta-BDE and octa-BDE), while deca-BDE production has been subsequently restricted exclusively to vehicle parts until 2036 [41,45]. A meta-analysis of eleven studies demonstrated that PCB-138 and PCB-153 are significant risk factors for CVD (OR = 1.35, 95% CI: 1.10–1.66; OR = 1.35, 95% CI: 1.13–1.62, respectively), whereas no association was observed for PCB180 or total PCB exposure [28]. More recently, a large-scale analysis of eleven studies extended these findings by showing robust positive associations between overall PCB exposure (OR = 1.56, 95% CI: 1.20–1.75), non-dioxin-like PCBs (OR = 1.33, 95% CI: 1.15–1.53), and CVD risk [46]. By contrast, the evidence base linking PBDE exposure to CVD or relevant cardiovascular risk factors is still sparse and inconclusive [47]. However, a recent cross-sectional study using data from NHANES 2005–2016 reported a 23% increased risk of CVD-including coronary heart disease, congestive heart failure, myocardial infarction, angina pectoris, or stroke-in association with higher serum levels of a PBDE mixture (PBDE-28, PBDE-47, PBDE-85, PBDE-99, PBDE-100, PBDE-153, and PBDE-154) (OR = 1.23, 95%CI: 1.03–1.47) [48]. Consistently, in U.S. adults, PBDE-209 and PBDE-153 were positively associated with total, remnant, and LDL-cholesterol after adjustment for all covariates, suggesting an increased risk of dyslipidemia and CVD [49].

The term dioxins refers to a heterogeneous group of structurally related, environmentally persistent chemicals generated as by-products of industrial activities, such as metal smelting and the combustion of chlorine-containing organic materials, and exerting toxicity through a common aryl hydrocarbon receptor (AhR)-mediated pathway [50,51]. This group includes polychlorinated dibenzo-*p*-dioxins (PCDDs), including 2,3,7,8-tetrachlorodibenzo-*p*-dioxin (TCDD), the most potent congener, together with polychlorinated dibenzofurans (PCDFs). Certain PCBs (dioxin-like PCBs) adopt a coplanar configuration of their biphenyl rings, which allows them to act similarly to dioxins [52]. An early systematic review of twelve longitudinal studies-ten of which were conducted in occupational settings-reported a dose-related increase in mortality from ischemic heart disease and, to a lesser extent, from overall CVD; however, the lack of adjustment for major cardiovascular risk factors limits the consistency and interpretability of these findings [51]. More recently, this evidence has been strengthened by a meta-analysis of ten studies, which found a 31% increased risk of CVD associated with exposure to dioxin-like PCBs (OR = 1.31, 95%CI: 1.10–1.57) [46]. Notably, in a representative sample of U.S. adults from NHANES 2003–2004, exposure to dioxins and furans was strongly associated with adverse lipid profiles-including increased LDL and total cholesterol, as well as triglycerides-supporting a link between dioxin exposure and CVD risk [53].

PAHs, generated by incomplete combustion from both natural sources (e.g., wildfires and volcanic activity) and anthropogenic sources (such as vehicle exhaust, residential heating, food grilling, tobacco smoke, and industrial processes), comprise several hundred compounds, although only 16 have been identified as priory pollutants, and seven as probable human carcinogens, by the U.S. Environmental Protection Agency [54,55]. Due to their chemical structure, consisting of two or more fused benzene rings, PAHs are characterized by high stability and hydrophobicity, with hydrophilicity and environmental mobility decreasing as the number of benzene rings increases [55,56]. Despite their environmental persistence, PAHs are extensively metabolized in humans into hydroxylated derivatives that are primarily excreted in urine; nevertheless, parent compounds, with reported half-lives of approximately 6–35 h, can also be detected in blood [57]. Current evidence links PAH exposure to the pathogenesis of atherosclerosis, cardiometabolic dysfunction, and arterial inflammation [56]. Furthermore, a recent systematic review of twenty studies conducted across diverse geographical regions reported consistent positive associations between PAH exposure and both CVD and established cardiovascular risk factors in occupational settings as well as in the general population [56]. A subsequent systematic review and meta-analysis including a total of nine prospective studies found a significant positive association between all classes of urinary PAH metabolites and both blood pressure (OR = 1.32; 95%CI: 1.19–1.48, *p* < 0.0001) and CVD (OR = 1.23; 95%CI: 1.16–1.30, *p* < 0.0001), while no association was observed with coronary artery disease [54].

In sum, accumulating evidence suggests a potentially important role of EDCs in increasing CVD risk, albeit with substantial heterogeneity in the strength of evidence across different chemical classes. Large, multicenter longitudinal studies with rigorous control of confounding factors, together with a deeper understanding of the underlying biological mechanisms, are warranted to confirm current findings and to inform regulatory strategies aimed at CVD prevention.

## 3. Gene–Environment Interaction: The Role of Genetic Polymorphisms in Susceptibility to EDC-Induced Cardiovascular Risk

Gene–environment interaction (GxE), whereby genetic background influences the biological response to external exposures, is now widely considered a major determinant shaping interindividual variability in susceptibility to environmental pollutants [58]. Previous evidence has highlighted the relevance of GxE in susceptibility to the effects of toxic elements related to multiple pathological conditions, including CVD [59].

In the context of EDCs, GxE mechanisms are being investigated to explain why comparable levels of exposure may lead to markedly different cardiovascular outcomes across individuals. As discussed in detail below, both genetic variants in candidate genes and polygenic risk profiles may influence key molecular and cellular pathways involved in oxidative stress regulation, xenobiotic metabolism and detoxification, hormone signaling, and lipid homeostasis, thereby modulating the cardiovascular effects of EDCs. Although the available evidence remains limited, emerging studies suggest that genetic variability may contribute to interindividual differences in susceptibility to EDC-induced CVD and related cardiometabolic risk factors.

To identify studies investigating gene–environment interactions between EDC exposure and cardiovascular outcomes, a literature search was conducted in PubMed up to 20 May 2026. Search terms included combinations of keywords related to endocrine-disrupting chemicals (‘bisphenol A’, ‘phthalates’, ‘PFAS’, ‘PCBs’, ‘PBDEs’, ‘dioxins’, and ‘polycyclic aromatic hydrocarbons’), genetic susceptibility (‘genetic polymorphism’, ‘single nucleotide polymorphism’, ‘SNP’, ‘polygenic risk score’, and ‘gene–environment interaction’), and cardiovascular outcomes (‘cardiovascular disease’, ‘hypertension’, ‘dyslipidemia’, ‘diabetes’, ‘obesity’, and ‘cardiometabolic risk’). To ensure comprehensive coverage of the available literature, no restrictions were applied regarding study design. Only articles published in English were considered, including original research articles, reviews, and meta-analyses. In addition, the reference lists of all selected articles were manually screened to identify further relevant studies.

The following paragraphs and Table 1 summarize the main findings currently available in terms of GxE in cardiovascular field.

### 3.1. Bisphenols

To date, only one study has directly examined the association between genetic polymorphisms and bisphenol-induced CVD: a case–control study conducted by Jiang et al. in 439 pairs of hypertensive-normotensive subjects showed that polymorphisms in genes involved in oxidative stress pathway, such as *CAT* rs769214 and rs4755374 (especially AC + CC genotype), *eNOS* rs1799983, and gene encoding for estrogen receptor 2, *ESR2* rs1256049, are associated with increased hypertension risk in relation to BPA exposure [22]. In particular, *CAT* encodes catalase, a major antioxidant enzyme involved in the maintenance of cellular redox balance, whereas *eNOS* regulates nitric oxide production and endothelial vascular homeostasis; therefore, alterations in these pathways exacerbate oxidative stress and endothelial dysfunction, two key mechanisms underlying BPA-induced cardiovascular damage.

Beyond ESR2, other hormone receptors involved in endocrine signaling pathways, such as estrogen receptor alpha, androgen receptor, and pregnane X receptor, which are expressed in cardiovascular tissues and participate in the regulation of vascular homeostasis, lipid metabolism, and inflammatory responses, may also be relevant in mediating the biological effects of EDCs and warrant further investigation in the context of GxE and cardiovascular risk [60].

However, polymorphisms may also indirectly impact cardiovascular risk induced by bisphenols, by promoting intermediate risk factors, such as obesity. Indeed, a polymorphism in the leptin receptor gene (*LEPR* rs9436303, G allele), which plays a crucial role in satiety regulation, resulted associated with increased body mass index in a population of adolescents and young adults (16–24 years), with a stronger effect in combination with high dietary bisphenol exposure [61]. In children (3–12 years), genetic variability in phase I and II metabolizing enzymes *CYP2C19* (rs4244285 G/A) and *GSTP1* (rs1695 A/G) has been associated with a twofold increased risk of overweight and obesity under conditions of long-term bisphenol exposure, although these associations showed borderline statistical significance. In the same study, the *INSIG2* rs7566605 C/G polymorphism, involved in lipid metabolism, was significantly associated with an almost threefold increased risk of overweight in subjects with short-term bisphenol exposure [62].

### 3.2. Phthalates

GxE involving phthalate exposure and cardiovascular risk remains largely unexplored, with the exception of a case–control study conducted by Wang et al. This study is particularly relevant, as sheds light on the association between maternal exposure to these toxicants during the periconceptional period and the risk of congenital heart disease in offspring, particularly septal defects in the presence of genetic variants in the *ABCB1* gene. This gene is essential for detoxification, since it encodes for P-glycoprotein (P-gp), the most abundant transporter in the human placenta, which actively effluxes xenobiotics out of cells. Notably, the study found that, although the T allele represents the polymorphic variant of *ABCB1* C3435T, increased susceptibility to congenital heart disease associated with maternal exposure to phthalates and alkylphenolic compounds (other EDCs), was observed in C allele carriers. Such effect may be explained by the lower *ABCB1*/P-gp expression observed in these individuals [63].

### 3.3. Per- and Polyfluoroalkyl Substances

In the case of PFAS exposure, only one study has reported significant findings in terms of GxE. In a cohort of 504 pregnant women, Kobayashi et al. demonstrated that maternal PFAS exposure significantly interacts with *PPARGC1A* (rs8192678) and *PPARD* (rs1053049, rs2267668) polymorphisms to alter circulating fatty acid profiles, particularly by reducing palmitic acid levels, thereby indicating a gene-dependent effect of PFAS on lipid metabolism [64]. Given the central role of *PPARGC1A* in oxidative stress responses [65] and of *PPARD* in fatty acid oxidation and lipid homeostasis [66], along with the well-established between circulating fatty acids and CVD risk [67], these interactions may contribute to PFAS-related cardiovascular effects.

### 3.4. Polychlorinated Biphenyls

To date, only two studies have investigated GxE related to PCB exposure and CVD-related conditions. Both studies based their analysis on the definition of polygenic risk scores (PRSs), rather than on individual polymorphisms, and examined their effects in the context of dysglycemia and dyslipidemia, well-established conditions linked to cardiovascular risk [68,69]. The first study, conducted by Tan et al. in 2676 participants from the Wuhan–Zhuhai cohort, applied a PRS constructed from 96 T2D-associated genetic variants. The authors reported a positive interaction between PCB-118 exposure and a high PRS, which was associated with increased fasting plasma glucose and a higher risk of developing T2D over a 6-year follow-up. These findings suggest that genetic predisposition may amplify the adverse effects of PCB exposure on glucose metabolism [70]. Using the same cohort, a PRS for dyslipidemia was subsequently developed. Individuals with a higher genetic predisposition exhibited an increased risk of hypercholesterolemia and hypertriglyceridemia when exposed to PCBs, further supporting the role of genetic background in modulating susceptibility to PCB-related metabolic disturbances [71].

### 3.5. Polybrominated Diphenyl Ethers

Evidence on GxE involving PBDEs remains scarce, with only one study currently available. In a recent investigation conducted by Hu et al. in a population of 871 participants, 3571 genetic variants were identified as interacting with PBDE exposure to influence dyslipidemia risk. Among those, the *FTO* gene rs9869609 G allele showed a stronger correlation with hypercholesterolemia, especially in the context of PBDE-47 exposure. Notably, the authors provided mechanistic insight by demonstrating that this SNP enhances the binding affinity of the BHLHE40 transcription factor, leading to downregulation of *SLC6A20* gene expression, a gene encoding a glycine transporter, and, consequently, increased cholesterol accumulation [72].

### 3.6. Dioxins

To date, no specific SNPs have been identified as genetic modifiers of the cardiovascular effects of dioxins or of dioxin-related cardiovascular risk factors. As a result, the potential contribution of genetic susceptibility to interindividual variability in cardiovascular responses to dioxin exposure remains largely unexplored.

### 3.7. Polycyclic Aromatic Hydrocarbons

Evidence of GxE involving PAHs and cardiovascular risk factors is currently limited to four studies. In the study of Fernández-Macías et al. in a population of women, the T allele rs1801133 polymorphism of *MTHFR* gene, encoding an enzyme involved in the metabolism of folates, was found to significantly interact with exposure to PAHs with remarkable effects on cardiovascular risk markers. Specifically, higher levels of asymmetric dimethylarginine (ADMA), a marker of endothelial dysfunction, and an increased atherogenic index of plasma were observed in carriers of this variant, with even greater elevations in the presence of high PAH exposure [19]. Similarly, a significant interaction has been reported between PAH exposure and the *PON1* gene rs662 polymorphism (G allele), also associated with increased ADMA levels. *PON1* encodes an antioxidant enzyme that prevents low-density lipoprotein (LDL) oxidation and thus protects against atherosclerosis; the coding-region polymorphism leads to an amino acid substitution that reduces enzymatic activity, potentially increasing cardiovascular risk [20]. The third study, based on data from the analysis of Wuhan–Zhuhai cohort employed a lipid-specific PRS. Individuals with a high PRS, when combined with elevated PAH exposure, exhibited increased levels of LDL-cholesterol, further supporting the role of genetic susceptibility in modulating PAH-related cardiometabolic risk [73]. The fourth study, conducted by Li et al. in a case–control population comprising 357 mothers of fetuses with congenital heart diseases and 270 control mothers, investigated the interaction between maternal PAH exposure and polymorphisms in genes involved in xenobiotic metabolism and PAH signaling pathways (AHR, CYP1A1, CYP1A2, CYP1B1, and CYP2E1). Significant gene–environment interactions were identified, particularly for the CYP1A2 rs4646425 and CYP2E1 rs915908 variants, which modified the association between maternal PAH exposure and the risk of congenital heart disease. These findings suggest that genetic variation in pathways regulating PAH metabolism and biological response may influence susceptibility to PAH-related cardiovascular effects during fetal development, thereby contributing to the risk of congenital heart defects [74].

**Table 1:** Genetic polymorphisms modifying cardiovascular risk induced by EDCs. Circles in the “Effect column” are green when SNPs may be correlated with a protective action, red with increased cardiovascular risk.

EDC	Reference	Pathway	Gene	SNP	Effect	
Bisphenols	[22]	Oxidative stress/antioxidant defense	CAT	rs 4755374 C allele	↑ hypertension risk	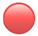
				rs769214 G/G	↑ hypertension risk	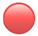
			eNOS	rs1799983 G/G		
		Hormone signaling and lipid metabolism	ESR2	rs1256049 C/C		
	[61]	Hormone signaling and lipid metabolism	LEPR	rs9436303 G allele	↑ BMI	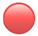
	[62]	Metabolism and detoxification	CYP2C19	rs4244285 G/A	2-fold ↑ risk of overweight and obesity	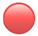
			GSTP1	rs1695 A/G		
		Hormone signaling and lipid metabolism	INSIG2	rs7566605 C/G	~3-fold ↑ risk of overweight	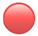
Phthalates	[63]	Metabolism and detoxification	ABCB1	rs1045642 C allele	↑ congenital heart disease risk	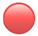
PFAS	[64]	Oxidative stress/antioxidant defense	PPARGC1A	rs 8192678 A allele	↓ circulating fatty acids	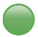
		Hormone signaling and lipid metabolism	PPARD	rs1053049 C allele, rs2267668 G allele		
PCBs	[70]	PRS			↑ fasting plasma glucose and risk of developing T2D	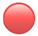
	[71]	PRS			↑ risk of hyperTC, hyperTG, and hyperLDL-C	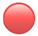
PBDEs	[72]	Hormone signaling and lipid metabolism	FTO	rs9869609 G allele	↑ hypercholesterolemia risk	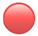
Dioxins	Not available					
PAHs	[19]	Metabolism and detoxification	MTHFR	rs1801133 T allele	↑ ADMA and AIP	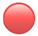
	[20]	Oxidative stress/antioxidant defense	PON1	rs662 G allele	↑ ADMA	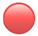
	[73]	Lipid-specific PRS			↑ LDL-C	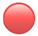
	[74]	Metabolism and detoxification	CYP1A2	rs4646425 T allele	↑ congenital heart disease risk	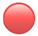
			CYP2E1	rs915908 A allele		

Abbreviations: ↓: decrease of; ↑: increase of; ADMA: asymmetric dimethylarginine; AIP: atherogenic index of plasma; BMI: body mass index; EDC: endocrine-disrupting chemicals; LDL-C: low-density lipoprotein cholesterol; OR: odds ratio; PAHs: polycyclic aromatic hydrocarbons; PBDEs: polybrominated diphenyl ethers; PCBs: polychlorinated biphenyls; PFAS: per- and polyfluoroalkyl substances; PRS: polygenic risk score; SNP: single-nucleotide polymorphism; T2D: type 2 diabetes; TC: total cholesterol; TG: triglycerides.

## 4. Conclusions and Emerging Perspectives

Accumulating evidence suggests that exposure to EDCs may contribute significantly to CVD development through complex biological mechanisms. Human biomonitoring studies have consistently demonstrated widespread exposure to PFAS, PCBs, PBDEs, PAHs, dioxins, phthalates, and bisphenols across populations, supporting growing concerns regarding their long-term cardiovascular consequences.

Importantly, interindividual variability in susceptibility to EDC-related cardiovascular effects cannot be explained solely by differences in exposure levels. Emerging evidence strongly suggests that genetic polymorphisms in pathways involved in oxidative stress responses, metabolism and xenobiotic detoxification, hormone signaling, and lipid homeostasis, may substantially modify cardiovascular responses to EDC exposure. Variants in these genes appear to influence the biological handling of environmental contaminants and the downstream activation of pathogenic pathways associated with cardiometabolic disease.

Specifically, variants in genes involved in antioxidant response, such as CAT, eNOS, and PON1, are associated with higher hypertension risk and enhanced vascular dysfunction under EDC exposure, particularly for bisphenols and PAHs. In parallel, variants in metabolism and detoxification-related genes, such as *GSTP1*, *CYP2C19*, *CYP1A2*, *CYP2E1*, *ABCB1*, and *MTHFR* appear to modulate susceptibility to cardiometabolic alterations and congenital heart defects under exposure to bisphenols, phthalates, and PAHs. Similarly, polymorphisms affecting lipid and metabolic homeostasis, including ESR2, FTO, LEPR, and INSIG2, are linked to hypertension, obesity, and dyslipidemia following exposure to PFAS, PBDEs, and bisphenols. Multiple genetic variants may affect susceptibility to hyperglycemia and hypercholesterolemia induced by PCBs. Conversely, no evidence has emerged from our analysis related to polymorphisms as modifiers of cardiovascular risk induced by dioxins.

Notably, all the considered studies of gene–environment interaction link EDCs exposure to an adverse cardiovascular outcome, except for the study of Kobayashi et al., where polymorphisms in PPARGC1A and PPARD genes decrease circulating fatty acids under PFAS exposure, effect that may lead to a reduced cardiovascular risk.

Although current findings are promising, the available evidence remains fragmented and is frequently limited by small sample sizes, heterogeneous study designs, differences in exposure assessment, variability in outcome definitions, and insufficient consideration of chemical mixtures. The reproducibility of several reported associations across independent populations also remains limited, while the extensive use of multiple-testing approaches may increase the likelihood of false-positive findings. In addition, potential publication bias toward statistically significant associations should be considered. These methodological limitations are further compounded by the fact that many studies have focused on single EDCs or individual polymorphisms, despite real-world exposure scenarios involving complex mixtures of contaminants interacting simultaneously with multiple genetic variants. Moreover, functional validation is still lacking for several identified susceptibility polymorphisms, limiting the ability to confirm the biological plausibility and mechanistic relevance of the observed associations. Finally, the lack of longitudinal studies integrating exposomic, genomic, and clinical data further weakens causal inference. Importantly, our literature review also identified emerging evidence adopting a broader approach based on polygenic risk scores, in which numerous susceptibility-associated polymorphisms were collectively evaluated in relation to the investigated disorder.

In line with these considerations, further research should prioritize large-scale prospective studies integrating human biomonitoring data with comprehensive genomic, transcriptomic, epigenetic, and metabolomic analyses. Particular attention should be devoted to GxE interactions during vulnerable developmental windows, including prenatal and early-life exposure. In addition, investigating the combined effects of multiple EDCs using mixture-based analytical approaches may better reflect real environmental exposure conditions and improve risk prediction models.

Another important perspective concerns the integration of precision medicine and environmental cardiology. Identifying individual genetic profiles associated with increased vulnerability to EDC exposure could contribute to personalized prevention strategies, early cardiovascular risk stratification, and targeted public health interventions. In this context, induced pluripotent stem cell-derived endothelial cells (iPSC-ECs) represent an innovative and promising experimental model for studying the vascular effects of environmental contaminants. Because iPSC-ECs retain the genetic background of the donor, they offer a unique opportunity to investigate how individual genetic susceptibility influences endothelial responses to EDC exposure [75]. These models may help clarify the molecular mechanisms underlying GxE interactions, including oxidative stress, inflammation, endothelial dysfunction, and altered vascular signaling pathways. Furthermore, iPSC-EC-based platforms could support the identification of early biomarkers of vascular damage and improve the development of personalized preventive and therapeutic strategies in environmental cardiology.

In conclusion, a deeper understanding of the interplay between environmental exposures and genetic susceptibility will be fundamental for advancing preventive cardiology and addressing the growing impact of environmental pollutants on cardiovascular health.

## Data Availability

No new data were created or analyzed in this study. Data sharing is not applicable to this article.

## Abbreviations

The following abbreviations are used in this manuscript:

ABCB1	ATP Binding Cassette Subfamily B Member 1
ADMA	Asymmetric Dimethylarginine
AhR	Aryl Hydrocarbon Receptor
AIP	Atherogenic Index of Plasma
BDE	Brominated Diphenyl Ether
BFRs	Brominated Flame Retardants
BHLHE40	Basic Helix-Loop-Helix Family Member E40
BMI	Body Mass Index
BPA	Bisphenol A
CAT	Catalase
CI	Confidence Interval
CYP	Cytochrome P450
CVD	Cardiovascular Disease
DOHaD	Developmental Origins of Health and Disease
EDC	Endocrine-Disrupting Chemical
eNOS	Endothelial Nitric Oxide Synthase
ESR	Estrogen Receptor
GST	Glutathione S-Transferase
GxE	Gene–Environment Interaction
HBM	Human Biomonitoring
INSIG2	Insulin Induced Gene 2
iPSC-ECs	Induced Pluripotent Stem Cell-Derived Endothelial Cells
LDL	Low-Density Lipoprotein
LDL-C	Low-Density Lipoprotein Cholesterol
LEPR	Leptin Receptor
MBzP	Monobenzyl Phthalate
MECPP	Mono-2-Ethyl-5-Carboxypentyl Phthalate
MEOHP	Mono-(2-Ethyl-5-Oxohexyl) Phthalate
MnBP	Mono-n-Butyl Phthalate
MTHFR	Methylenetetrahydrofolate Reductase
NHANES	National Health and Nutrition Examination Survey
OR	Odds Ratio
PAH	Polycyclic Aromatic Hydrocarbon
PBDE	Polybrominated Diphenyl Ether
PCB	Polychlorinated Biphenyl
PCDDs	Polychlorinated Dibenzo-p-Dioxins
PCDFs	Polychlorinated Dibenzofurans
PFAS	Per- and Polyfluoroalkyl Substances
PFHxS	Perfluorohexanesulfonic Acid
PFOA	Perfluorooctanoic Acid
PFOS	Perfluorooctane Sulfonic Acid
P-gp	P-Glycoprotein
POP	Persistent Organic Pollutant
PPARD	Peroxisome Proliferator-Activated Receptor Delta
PPARG	Peroxisome Proliferator-Activated Receptor Gamma
PPARGC1A	PPARG Coactivator 1 Alpha
PRS	Polygenic Risk Score
RR	Relative Risk
SLC6A20	Solute Carrier Family 6 Member 20
SNP	Single-Nucleotide Polymorphism
T2D	Type 2 Diabetes
TCDD	2,3,7,8-Tetrachlorodibenzo-p-Dioxin
TC	Total Cholesterol
TG	Triglycerides
